# Diversity, transfer potential, and transcriptional activity of virus‐carried antibiotic resistance genes in global estuaries

**DOI:** 10.1002/imo2.70074

**Published:** 2026-01-07

**Authors:** Xiao‑Qing Luo, Xiyang Dong, Jia‑Ling Li, Ji‐Cheng Yao, Ziqi Peng, Qi Li, Zi‐Wen Yang, Yang Yuan, Wen‑Jun Li, Pandeng Wang

**Affiliations:** ^1^ School of Pharmacy (Shenzhen University Medical School), College of Life Sciences and Oceanography, and Institute for Advanced Study, Shenzhen University Shenzhen China; ^2^ State Key Laboratory of Biocontrol, Guangdong Provincial Key Laboratory of Plant Stress Biology, School of Life Sciences Sun Yat‐Sen University Guangzhou China; ^3^ Key Laboratory of Marine Genetic Resources, Third Institute of Oceanography Ministry of Natural Resources Xiamen China; ^4^ Southern Marine Science and Engineering Guangdong Laboratory (Guangzhou) Guangzhou China; ^5^ Institute of Marine Biotechnology and Bio‐Resource Utilization, College of Oceanography Hohai University Nanjing China

**Keywords:** antibiotic resistance genes, estuarine ecosystem, global warming, virus

## Abstract

Estuaries, the transition zones between terrestrial and marine ecosystems, are vital hotspots for antibiotic resistance genes (ARGs) due to substantial antibiotic pollution. Although viruses have been considered as reservoirs and vectors of ARGs in various environments, the composition, transmission, and ecological drivers of virus‐carried ARGs (vARGs) in estuarine ecosystems remain poorly understood. Here, we constructed a Global Estuarine Virome (GEV) dataset by recovering 136,443 viral operational taxonomic units (vOTUs) from 534 worldwide estuarine metagenomes. Based on this GEV dataset, we identified 282 (temperate: 217; lytic: 65) vOTUs that carried 462 ARGs. These ARGs encompassed 108 ARG subtypes conferring resistance to 13 antibiotic classes, with their richness and abundances in estuaries being directly and indirectly correlated with changes in temperatures and coastal human activities. Network approach further revealed temperature‐dependent vARG transfers between viruses and prokaryotes. Moreover, ARG‐carrying vOTUs could target abundant prokaryotic taxa (e.g., *Rhodobacteraceae* and *Flavobacteriaceae*) and potential pathogens, including *Acinetobacter venetianus* and *Pseudomonas marginalis*. Notably, leveraging 188 estuarine metatranscriptomes, we found that over half of the vARGs (51%) were active in estuaries, and the expression levels of vARGs were sometimes comparable to their prokaryotic counterparts. The relative contribution of viruses to ARG expression also increased under warmer conditions. Overall, although the ARGs were only detected in a small fraction (0.2%) of the estuarine vOTUs, this study reveals that the potential risks of vARGs in estuarine ecosystems warrant attention, especially in the context of global warming and socioeconomic development. This underscores the necessity of incorporating vARGs into future surveillance and management of estuaries.

## Highlights

- Estuarine viruses, particularly temperate viruses, carried abundant and diverse antibiotic resistance genes (ARGs), and over half of the virus‐carried ARGs (vARGs) were actively expressed in estuarine environments.
- vARG‐carrying viruses targeted a wide range of prokaryotes and likely facilitated horizontal gene transfer of vARGs between viruses and prokaryotes, supported by sequence similarity and minimal evolutionary distance analyses.
- Elevated temperatures and coastal human activities could significantly increase the richness, abundance, transmission risk, and relative expression of estuarine vARGs.

## Graphical abstract

Estuaries are vital hotspots for antibiotic resistance genes (ARGs) due to substantial antibiotic pollution. Although viruses have been proposed as key reservoirs and important disseminators of ARGs in environments, their contribution to the estuarine antibiotic resistome remains largely unknown. Here, by constructing a Global Estuarine Virome dataset from 534 worldwide estuarine metagenomes, we found that estuarine viruses, particularly temperate viruses, carried abundant and diverse ARGs, and over half of the virus‐carried ARGs (vARGs) were actively expressed in estuarine environments. Further analysis based on sequence similarity and minimal evolutionary distance revealed that vARG‐carrying viruses targeted a wide range of prokaryotes and likely facilitated horizontal gene transfer of vARGs between viruses and prokaryotes. Importantly, elevated temperatures and coastal human activities could significantly increase the richness, abundance, transmission risk, and relative expression of estuarine vARGs. These findings provide important implications for managing the estuarine antibiotic resistome.

## 1 INTRODUCTION

Estuaries, as important land‐sea transition zones, not only nourish about 70% of the world's largest cities but also are hotspots for human activities [1]. In recent decades, estuaries have emerged as critical sinks for antibiotics due to rising antibiotic usage and human activities (e.g., sewage discharge and aquaculture effluent) [2, 3]. For instance, over 3000 tons of antibiotics were released into the Yellow River Estuary and the Yangtze River Estuary during one year [2], and more than 193 tons of antibiotics are annually discharged into the Pearl River Estuary [4]. Moreover, the high concentration of amoxicillin in the Tejo Estuary (median concentration of 70.48 ng/L) exceeded the safety threshold by over 30 times [3]. The accumulation of antibiotics in estuaries exerts persistent selective pressure on microbial communities, increasing the abundance and transmission of antibiotic resistance genes (ARGs) [5] and thereby posing threats to human health and clinical treatment efficacy [6, 7, 8]. Recently, despite growing public concerns about the severe ARG pollution in estuaries, our scientific understanding remains skewed heavily toward prokaryote‐carried ARGs (pARGs) [9, 10]. In addition to pARGs, virus‐carried ARGs (vARGs) may be another non‐negligible component of the estuarine antibiotic resistome. Additionally, viruses can transfer their genes (including ARGs [11]) into host genomes, and they are abundant and exhibit strong persistence in environments [12], making them ideal genetic vehicles for ARG transmission [13]. However, research on vARGs has largely lagged behind that of their prokaryotic counterparts, and revealing the composition, transmission potential, and environmental drivers of vARGs would give us a more thorough comprehension of the estuarine antibiotic resistome.

With the rapid development of metagenomics and viromics, numerous ARGs conferring resistance to lactamase, quinolone, and vancomycin antibiotics have been detected in viral genomes (including phages and giant viruses [14]) across diverse environments, including seawater [15], rivers [16], soils [17, 18], animal guts [19], and engineered systems [20, 21, 22]. Moreover, the functionality of some vARGs that are related to beta‐lactam (*bla*_HRV‐1_ and *bla*_HRVM‐1_), streptomycin (*aadA2*), chloramphenicol (*catII*), and trimethoprim (*dfrC*) resistance was confirmed using heterologous expression technique [14, 16, 23]. These previous findings indicate that viruses are potential reservoirs of ARGs. However, despite the high antibiotic selective pressure in estuaries [5], the richness and abundance of vARGs have rarely been characterized, leaving them as unexplored aspects of the estuarine antibiotic resistome. More importantly, although vARGs have been found transcriptionally active in numerous habitats [23], the relative contributions of viruses to ARG expression in estuaries are not yet known, which hampers our full understanding of the viruses' roles in estuarine antibiotic resistance.

Virus‐mediated transduction is one of the mechanisms facilitating ARG spread [13, 24]. Unlike transformation and conjugation, viruses transmit their ARGs within viral capsids and do not require direct cell‐to‐cell contact [25]. Therefore, vARGs can be transferred between bacteria that are present at different times and places. Additionally, given that some viruses usually have broad host spectrums [11, 26, 27, 28], virus‐mediated gene transfer is not restricted to microorganisms of the same species but may occur between different species [11], genera [29], or families [30]. In this context, exploring the transmission range and capacity of vARGs is essential for effectively managing the spread of antibiotic resistance, especially in the antibiotic‐polluted estuaries. Along with the development of molecular and bioinformatic techniques, recent studies have shed light on vARG transfers in diverse habitats using DNA sequencing and virus‐host prediction [18, 31], DNA homology analysis [32], as well as transductomics [33]. Nevertheless, we still know little about the characteristics of vARG transfers in estuarine environments.

Another significant challenge in managing estuarine antibiotic resistance is the limited understanding of environmental drivers that influence vARG characteristics (i.e., richness, abundance, transmission, and expression). Several factors, including geographic locations, socio‐economic status, human activities, and seasonality, have been proposed to structure the vARG richness and abundance in agricultural soil [18], urban sewage [34], and river ecosystems [35]. In addition, the transduction of ARGs in soil was found to be affected by antibiotics, bacterial compositions, climate (e.g., temperature), and physicochemical conditions (e.g., organic matter and heavy metals) [13]. Exploring the drivers that shape vARG characteristics is crucial for predicting how global change might alter their ecological risks, yet the factors affecting vARGs in estuaries are still understudied.

To address these knowledge gaps, we collected and sequenced 60 metagenomes and 38 metatranscriptomes from the Pearl River Estuary, and retrieved 474 metagenomes, 150 metatranscriptomes, and relevant environmental variables from public databases. This estuarine dataset encompassed 551 water, 166 sediment, and 5 mat samples from 74 estuaries across nine countries. Utilizing this dataset, we profiled the viruses and their ARGs to address two main questions: (i) how vARGs contribute to the estuarine antibiotic resistome in terms of diversity, abundance, transmission, and expression, and (ii) what factors influence these vARG characteristics?

## 2 RESULTS

### Overview of the global estuarine virome (GEV) dataset

To investigate viruses inhabiting estuarine environments, we constructed a GEV dataset (Figure S1) by recovering DNA viral genomes from 534 estuarine metagenomes (~15 Tb) that were distributed across three continents and eight countries (Figure 1A and Table S1). In total, we recovered 146,207 viral contigs with length ≥10 kbp, which were then clustered into 136,443 viral operational taxonomic units (vOTUs; Table S2) to represent approximately species‐level taxonomy. To explore the genomic novelty of viruses in our GEV dataset, we clustered GEV with viral contigs from other published datasets, including the Global Ocean Viromes (GOV) 2.0 [36], wetland viral genomes [37], Global Soil Virome dataset (GSV) [38], land use virome dataset (LVD) [39], and Viral RefSeq r217 database [40], by using gene‐sharing network analysis. We found that more than 70% of the GEV's vOTUs did not share clusters with other databases, suggesting that a large number of novel viral genera existed in the estuarine samples (Figure 1B). The rising species accumulation curves further supported that the diversity of estuarine viromes remains largely unexplored (Figure 1C). Furthermore, only 12.8% of the estuarine vOTUs could be classified into viral families, and those classified vOTUs were mainly affiliated to the class *Caudoviricetes*, encompassing family *Kyanoviridae*, *Autographiviridae*, *Demerecviridae*, and *Zobellviridae* (Figure 1D). Taken together, these results indicate that estuarine viromes are novel and diverse.

**FIGURE 1:**
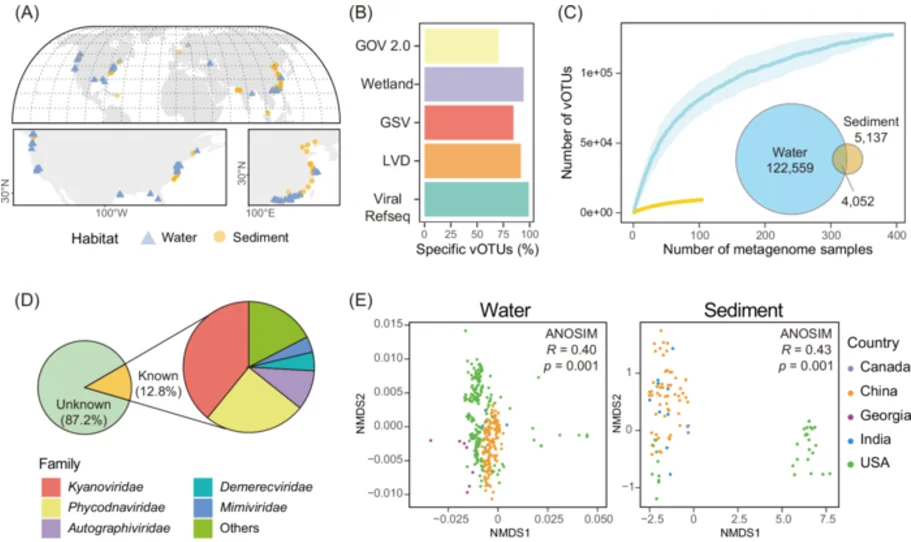
Overview of the global estuarine virome (GEV) database. (A) The map showing the sampling locations and sample types of the metagenomes used in this study. (B) Percentages of specific viral operational taxonomic units (vOTUs) within the GEV compared with five published datasets: Global Ocean Viromes (GOV) 2.0, wetland viral genomes (wetland), Global Soil Virome dataset (GSV), land use virome dataset (LVD), and Viral RefSeq r217 database (Refseq). (C) Species accumulation curves of vOTUs in the water (blue) and sediment (yellow) metagenomes. Dots represent the average number of vOTUs for all random sampling (100 times) of a given number of metagenomes, and error ribbons represent the 95% confidence intervals. Venn diagram displaying the number of unique and shared vOTUs in water and sediment habitats. (D) Taxonomic compositions of vOTUs in the GEV. The pie chart on the left indicates the proportion (%) of vOTUs with assigned family‐level taxonomy (known), and the pie graph on the right shows the relative frequency (%) of viral families. (E) Non‐metric multidimensional scaling (NMDS) of viral communities based on Bray‐Curtis dissimilarity. The significant differences of viral communities among countries were verified by analysis of similarities (ANOSIM; 999 permutations).

We further observed that the majority of estuarine vOTUs were endemic to either water (122,559 vOTUs) or sedimentary (5137 vOTUs) environments, with only 4052 vOTUs being present in both habitats (Figure 1C). Similar results were obtained after rarefying the water samples (Figure S2A). Moreover, the compositions of estuarine vOTUs exhibited significant differences across countries in both water and sediment habitats (Figures 1E and S2). The country‐dependent vOTU compositions might be related to the changes of prokaryotic community, ambient temperature, and the GDP per capita of the nearby region (Mantel's *r* > 0.25, *p* < 0.05; Figure S3). In addition, the compositions of estuarine viral communities in water were also correlated with salinity, while those in sediment were associated with the concentration of ferrous ions (Figure S3).

### Estuarine viruses, especially temperate viruses, carry diverse vARGs

A total of 282 ARG‐carrying vOTUs were identified from the GEV (Table S3), representing approximately 0.2% of the total estuarine vOTUs. This percentage was much higher than that in marine (0.01%) environments (Table S4), which may be attributed to the severe antibiotic pollution in estuaries [2, 3]. Moreover, predicted estuarine virus‐carried ARGs (vARGs; *n* = 462) were highly diverse, encompassing 108 subtypes of ARGs that could confer resistance to 13 classes of antibiotics through six distinct resistance mechanisms (Figure 2A and Table S5). Among all ARG‐carrying vOTUs, 33%, 20% and 6% of them encoded *msbA*, *novA*, and *patB*, respectively. Consistent with estuarine/coastal prokaryotic antibiotic resistome [10, 41], the predominant types of estuarine vARGs were related to nitroimidazole, multidrug, aminocoumarin, β‐lactam, and tetracycline, with antibiotic efflux and antibiotic inactivation being the most commonly annotated resistance mechanisms (Figure 2A). Notably, 25 vARG subtypes like *acrB*, *bacA*, and *MexH* have also been ranked as high health risk (Q1) according to their combined effects of human accessibility, mobility, pathogenicity, and clinical availability [42]. Furthermore, these diverse vARGs were widely distributed in global estuaries (Figure 2B), and they could contribute more than 10% of the total ARG abundance (sum of the abundance of both vARGs and pARGs) in 17 samples, indicating that vARGs composed an important part of the antibiotic resistome in estuaries.

**FIGURE 2:**
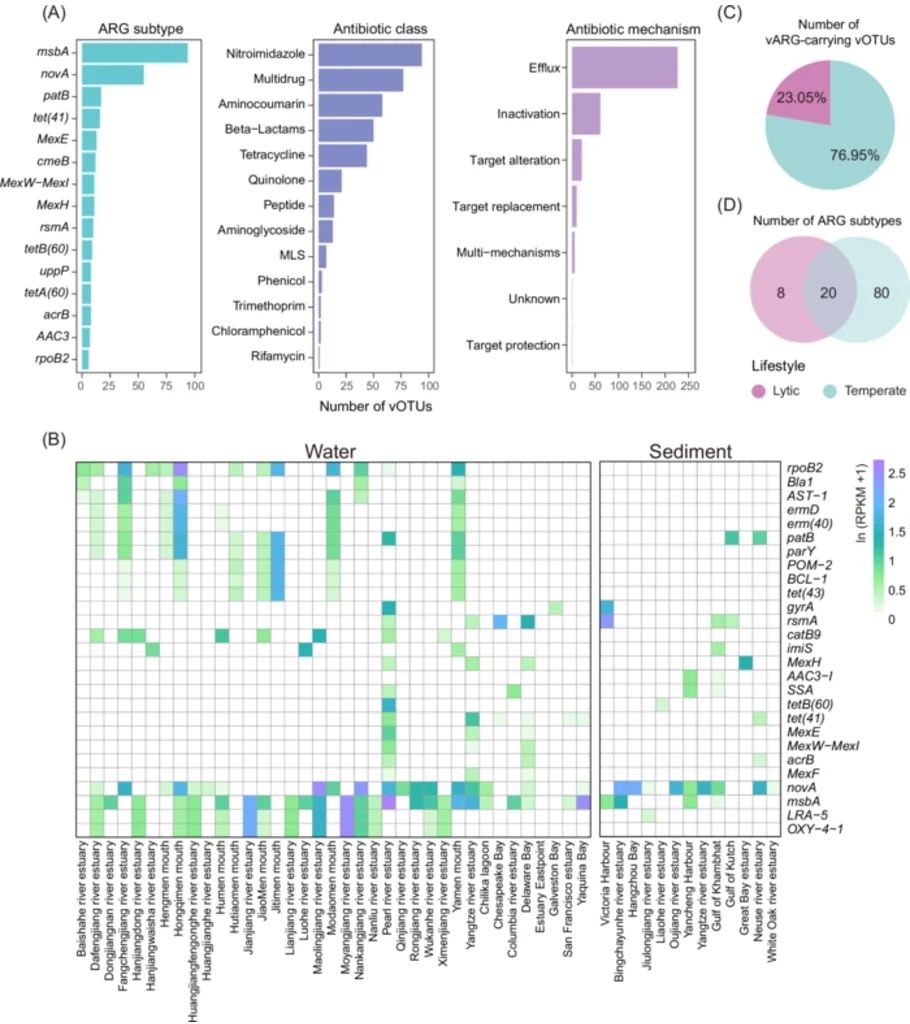
Abundant and diverse virus‐carried antibiotic resiatance genes (vARGs) in estuarine environments. (A) Numbers of viral operational taxonomic units (vOTUs) that carry different ARG subtypes, confer resistance to different antibiotic classes, or utilize different antibiotic mechanisms. (B) Abundance of each vARG subtype in different estuaries. Values are ln (RPKM + 1) transformed. (C) Proportion of lytic and temperate ARG‐carrying vOTUs. (D) The number of unique and shared ARG subtypes between different viral lifestyles. Eight vARG subtypes (including *CARB‐55*, *CIM‐1*, *dfrB6*, *dfrB9*, *EFM‐1*, *rpoB2*, *WUS‐1*, and *ZOG‐1*) are unique to lytic viruses.

We further performed viral lifestyle prediction and found that approximately 77% (217/282) of the total ARG‐carrying vOTUs were predicted to be temperate viruses (with lysogeny‐specific features or prophage signals, Figure 2C), which were also the dominant ARG‐carrying viruses in 78% (215/277) estuarine samples (Figure S4). Given the ability of temperate viruses to insert themselves into host chromosomes as prophages without causing cell lysis [43], the fitness advantage provided by vARGs for hosts would be more beneficial for temperate viruses, especially in antibiotic‐polluted environments. Moreover, temperate viruses exhibited higher ARG diversity than their lytic counterparts (temperate: 100 types vs. lytic: 28 types), with 80 vARG subtypes exclusively being detected in temperate viral communities (Figure 2D).

### Estuarine vARGs target a broad range of hosts and exhibit significant transmission potential

Utilizing multiple methods (see “Methods”), we successfully linked 4841 vOTUs to their potential prokaryotic hosts, including seven archaeal and 64 bacterial phyla (Table S6). The majority (83%) of ARG‐carrying vOTUs were assigned to potential hosts, which spanned two archaeal and 36 bacterial phyla, with Pseudomonadota (*Rhodobacteraceae*), Bacteroidota (*Flavobacteriaceae*, *Crocinitomicaceae*, and *Cyclobacteriaceae*), and Actinomycetota (*Nocardioidaceae*) being the most frequently predicted prokaryotic hosts (Figure 3A and Table S6). We found that vOTUs with higher vARG density (the number of vARGs was normalized by the sequence length of each vOTU) tended to interact with more prokaryotic taxa (Logistic regression, *p* < 0.001; Table S7). Moreover, three vOTUs that carried efflux pump genes for nitroimidazole, aminocoumarin, and tetracycline were linked to archaea (Thermoplasmatota and Thermoproteota), potentially conferring multidrug resistance to these archaeal hosts. Furthermore, viruses carrying vARGs against multidrug, aminoglycoside, beta‐lactams, quinolone, tetracycline, peptide, and nitroimidazole could target over a hundred prokaryotic families (Figure 3B), suggesting that the potential hosts of these vARGs were diverse.

**FIGURE 3:**
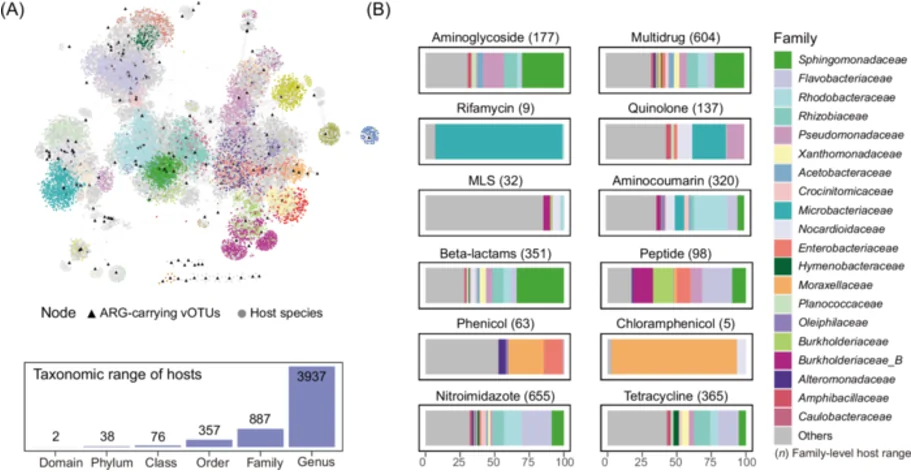
Predicted virus‐host linkages of the viral operational taxonomic units (vOTUs) carrying antibiotic resistance genes (ARGs) in the GEV. (A) Network illustrates the virus‐host connections of ARG‐carrying vOTUs in the GEV. Black triangles in the network represent ARG‐carrying vOTUs, while circles with different colors represent host species within different families. Bar plot indicates the taxonomic ranges of all predicted hosts in the network at various levels. (B) Number in brackets indicates the family‐level host range of all vOTUs that carry a specific ARG type. The bars denote the relative frequencies of vOTUs that infect different host families among all vOTUs that carry the specific type of ARG. Figures A and B share the same legend. GEV, global estuarine virome.

Virus‐mediated transduction is considered one of the routes for disseminating ARGs among prokaryotic hosts [44]. Here, we revealed 443 potential ARG transfers involving 178 vARGs, 112 vOTUs, and 235 different host species based on minimal evolutionary distances [32] (no less than 90% identity and 90% coverage; Figure 4 and Table S8). Among the ARG transfers, multidrug and nitroimidazole resistance genes were the most frequently transmitted (*n* = 124 and 121, respectively). A total of 39 vOTUs carrying nitroimidazole resistance genes were found to be able to transfer ARGs across 21 host families, including estuarine abundant taxa such as *Flavobacteriaceae* (3.2% average relative abundance across all estuarine prokaryotic communities) and *Sphingomonadaceae* (0.5%). Additionally, 58 transfer events of aminocoumarin resistance genes were observed between 23 vOTUs and 47 host species that were predominantly affiliated with *Oleiphilaceae* and *Sphingomonadaceae*. Meanwhile, the transfers of beta‐lactams, tetracycline, and peptide resistance genes were primarily associated with *Sphingomonadaceae*, *Devosiaceae*, and *Enterobacteriaceae*, respectively. Furthermore, we found that two or more different ARGs were involved within one specific “virus‐host” linkage (*n* = 67), or some viruses carrying different ARGs were linked to the same host, potentially facilitating the emergence of multidrug‐resistant bacteria. These results suggest a substantial potential for virus‐mediated ARG dissemination in estuaries.

**FIGURE 4:**
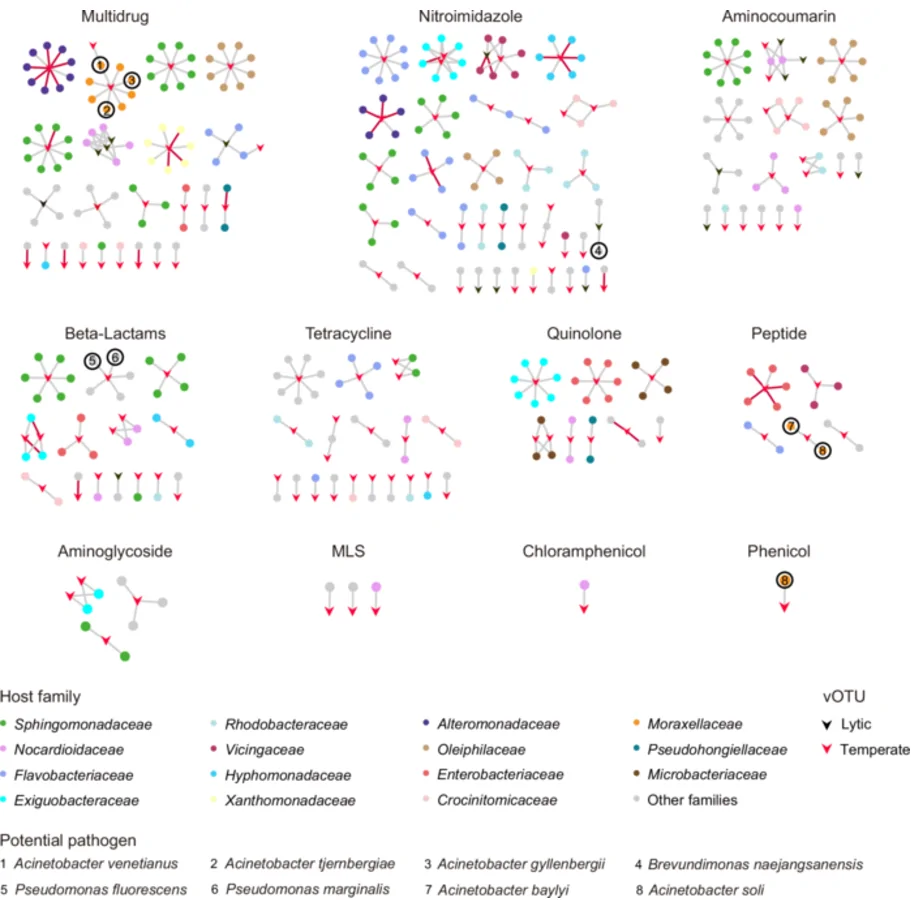
Virus‐mediated antibiotic resistance genes (ARG) transfers in the estuarine environments. Networks showing the transfer trajectories of ARGs between viral operational taxonomic units (vOTUs) and their hosts, which were identified based on the minimal evolutionary distances. Dots with different colors represent the ARG‐carrying vOTUs (“V” shape) and their predicted host species (circle). Gray and red connecting lines denote the transfer of single ARG and multiple ARGs (defined as at least two ARG subtypes) within one virus‐host linkage, respectively. MLS, macrolides, lincosamides, streptogramins.

According to the taxonomy annotations of host species, we found that potential pathogens [45] were involved in nearly 2.3% of ARG transfer events (*n* = 10; Figure 4 and Table S8). For example, temperate viruses can mediate the dissemination of multidrug (*adeI*), peptide (*uppP*), and phenol (*catB9*) resistance genes among clinically relevant pathogens, including *Acinetobacter venetianus* (0.03% average relative abundance across all estuarine prokaryotic communities), *Acinetobacter soli* (0.002%), and *Acinetobacter gyllenbergii* (0.0002%). Moreover, viruses carrying *ampC* gene can endow *Pseudomonas marginalis* (0.03%) and *Pseudomonas fluorescens* (0.01%) with the capability to withstand beta‐lactam antibiotics, potentially posing more severe threats to the ecosystem and human health.

### vARGs are actively expressed in estuarine environments

To examine whether vARGs were active in natural estuarine environments, we mapped high‐quality metatranscriptomic reads to both vARGs and pARGs using strict thresholds (100% coverage and 100% identity [46]). Over half (51%) of the vARGs were active in the estuaries (Table S9). Among them, the *msbA* gene was identified as the most active vARG, with high expression levels (ranked top five within all vARGs) in 22% (41/188) of all metatranscriptome samples. Since *msbA* gene can protect bacteria by exporting nitroimidazole compounds via efflux [47], the active viral‐carried *msbA* could offer survival advantages to their hosts under antibiotic stress, thereby enhancing viral fitness. Furthermore, we found that multiple vARGs were expressed simultaneously in almost half of the samples, especially those from the Pearl River Estuary and Chesapeake Bay. Given the prevalence of active vARGs in estuaries, we further quantified the relative contributions of viruses to ARG expression and found that over 5% of total ARG expression was contributed by vARGs in three samples (Figure 5 and Table S10). On some occasions, the relative expressions of some vARGs, such as *SSA*, *AcrF*, *adeF*, *adeJ*, *LRA‐3*, *and tet(A)*, exceeded their prokaryotic counterparts (Figure 5 and Table S11). Notably, the viruses carrying *adeJ* gene primarily infected *Acinetobacter*, which is a well‐known genus that contains various pathogenic species (Table S6). Considering that the ARG‐carrying temperate viruses might persist in their host for a long time, these highly expressed multidrug‐resistant vARGs in pathogenic species may contribute to the emergence of “super pathogens.” In summary, these results demonstrated that vARGs were actively expressed in estuarine environments and made a non‐negligible contribution to the estuarine antibiotic resistome.

**FIGURE 5:**
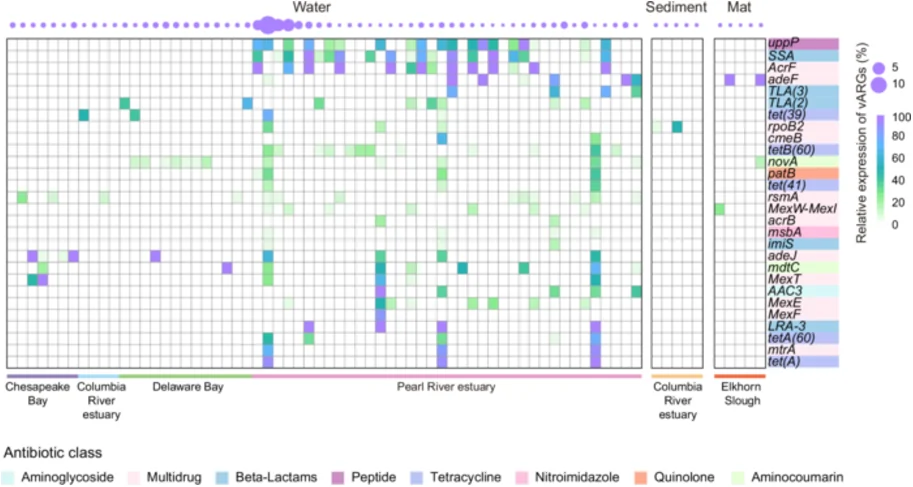
Contributions of virus‐carried antibiotic resistance genes (vARGs) to the overall expression of estuarine antibiotic resistome. Heatmaps of the relative expression of specific vARG (expression of specific vARG/total expression of the specific ARG: vARG + pARG) across different estuarine samples. Only vARGs that were actively expressed in more than two different estuarine samples are shown in the heatmap. Circle at the top of the heatmap indicates the relative expression of all vARGs in each sample, which was calculated as the ratio of total expression of vARGs to the sum expression of all vARGs and pARGs. pARGs, prokaryote‐carried ARGs.

### Elevated temperature and human activities may increase the risks of vARGs

Estuarine vARGs exhibited remarkable spatial heterogeneity (see Supplementary results and Figures S5–S8). Correlation and random forest analyses showed that the changes of vARG richness, abundance, expression, and transmission (i.e., number of vARG transfer types, transfer range, and potential transfer risk) across estuaries were primarily correlated with biotic factors, including the richness of pARGs and the whole viral (vTax) and prokaryotic (pTax) communities, temperature, latitude (mainly mirroring the change of temperature), and human activities (i.e., population density and urban ratio, Figure 6A, Figures S9, S10, and S11A). Additionally, vARG richness and transmission in water samples were positively correlated with precipitation and sewage emission, whereas significant positive correlations were observed between vARG richness and concentration of Fe^2+^ in sedimentary environments (Figure S9).

**FIGURE 6:**
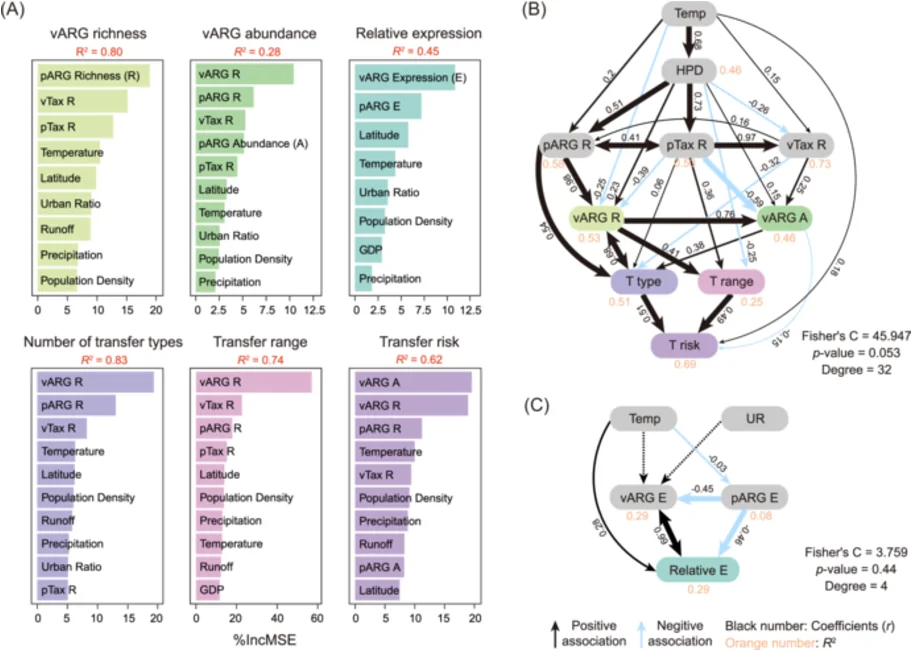
Drivers of the richness, abundance, expression, and transmission of virus‐carried antibiotic resistance genes (vARGs) in estuarine water. (A) Random forest analysis determined the relative importance of variables (only show the top ten) influencing vARG richness, abundance, expression, and transmission (i.e., number of vARG transfer types, transfer range, and transfer risk). *R* ^*2*^ (pseudo) represents the total variance that could be explained by the selected environmental variables. pTax, the whole prokaryotic communities; vTax, the whole viral communities. (B, C) Structural equation models show the potential direct and indirect effects of biotic and abiotic variables on the transmission risk and expression of vARGs. Solid black and blue arrows indicate significant positive and negative associations, respectively. A black dashed line denotes a non‐significant positive correlation. The standardized path coefficients (*r*, black number) are shown along the paths, and *R* ^*2*^ (orange number) represents the explained variance for every dependent variable. Temp, air temperature; HPD, human population density; UR, urban ratio; R, richness; A, abundance; T, vARG transfer; E, expression.

We further used structural equation modeling (SEM) to discern the potential causality among biotic factors, ambient temperature and human activities, and found that temperature and human activities exhibited both direct and indirect effects on vARGs (Figure 6B,C and Figure S11B). For example, in water environments, elevated temperature may directly increase the transfer risks of vARGs and indirectly via affecting human population density, pARG and pTax richness, which in turn increased vARG richness, transfer types and ranges (Figure 6B). Elevated temperature may also increase the relative contributions of vARGs to the overall expressions of estuarine ARGs (Figure 6C). While in sedimentary environment, temperature and prokaryotic richness indirectly affected vARG richness and abundance via the vTax richness and abundance (Figure S11B). Taken together, our findings highlighted the potential effects of temperature and human activities on the risks of vARGs, advancing our understanding of the complex interactions among viruses, hosts, and the environment.

## 3 DISCUSSION

Severe antibiotic pollution caused by anthropogenic activities is exacerbating the enrichment and dissemination of ARGs in estuarine environments. Viruses as potential reservoirs and disseminators of ARGs in natural environments are understudied in estuaries. In this study, leveraging the available estuarine multi‐omics datasets, we systematically examined the richness, abundance, transmission, and expression of estuarine vARGs at a global scale. We found that vARGs were diverse, transferable, and actively expressed in estuaries, and these features were sensitive to changes in ambient temperature and human activities.

Viruses, as the most abundant biological entities on earth, have been reported as important vehicles for ARGs in diverse environments, including soil, marine, and engineered systems [15, 18, 31, 48]. Consistent with previous studies [18, 49], we found that the ARG‐carrying vOTUs represented a low prevalence (about 0.2% in the overall GEV dataset) in estuarine environments. This prevalence may be somewhat underestimated due to highly stringent parameters for vARG detection. Furthermore, it should be noted that the ARG‐carrying rate of estuarine viruses was much higher than that in marine (0.01%) environments (Table S4). Additionally, vARGs were diverse and widespread in estuaries (Figure 2B), and even contributed more than 10% of the total ARG abundance in some samples. One possible reason could be that human activities, especially the overuse and abuse of antibiotics in coastal domestic settlements, livestock facilities, and aquacultures, bring large amounts of antibiotics into estuaries via rivers and streams [5, 50], exerting intense antibiotic selective pressure on prokaryotic microbes. This pressure could increase the fitness advantage of ARG‐carrying microbes, as well as the viruses that infect these hosts, thereby increasing the likelihood of viruses acquiring ARGs. This potential was, to some extent, confirmed by our structural equation model that showed human activities could increase the richness and abundance of vARGs by affecting pARGs (Figure 6B). The strong associations between vARGs and pARGs were also supported by the fact that the prevalent ARGs in the communities of viruses and prokaryotes were both multidrug resistance genes [9, 10]. More importantly, we found that the expression levels of vARGs were sometimes comparable to their prokaryotic counterparts, suggesting the significant contributions of vARGs to conferring resistance for prokaryotic hosts in estuarine environments, although further experiments are needed to verify these.

As expected, most of the ARG‐carrying vOTUs in estuaries were temperate viruses (Figure S4), consistent with previous studies in landfill leachate treatment plants, a landscape lake, and global human‐impacted environments [20, 21, 23]. This phenomenon could be attributed to the distinct lifestyle characteristics of temperate and lytic viruses. Unlike lytic viruses that usually immediately hijack host metabolism for progeny production after entering the host cells, temperate viruses often incorporate their genomes into hosts' chromosomes and replicate alongside their hosts as symbionts [51]. In this context, the fitness advantage provided by vARGs for hosts would be more beneficial for temperate viruses in antibiotic‐polluted environments (e.g., estuaries), which was partially supported by the finding that most actively expressed vARGs derived from temperate viruses (Figure S12). Additionally, temperate viruses can be viewed as “dangerous molecular time bombs,” capable of bursting their hosts by entering into lytic cycle due to environmental changes (e.g., anomalously high temperatures) [52]. Thus, the proliferation and transmission risks of ARGs carried by temperate viruses would be contingent upon environmental factors. As global climate change intensifies [53], the ecological risk posed by temperate virus‐encoded ARGs may become more pronounced. However, it is important to acknowledge that the lack of estuarine viromic data at present may have biased the identified viral sequences toward intracellular viruses, potentially leading to an underestimation of the ecological roles of lytic viruses in the estuarine antibiotic resistome.

Diverse vARGs can be transferred among prokaryotic hosts via virus‐mediated transduction. The transfer risks of vARGs could be higher when they are carried by viruses with broader host ranges [54]. In this study, we found that the ARG‐carrying viruses could not only interact with a broader range of prokaryotic taxa than viruses without ARGs, but also target abundant prokaryotic taxa (e.g., *Rhodobacteraceae* and *Flavobacteriaceae*) across estuaries, indicating a significant risk of virus‐mediated ARG transmissions in the estuarine environment. Furthermore, diverse ARG transfers between viruses and their host genomes were further confirmed by network analyses (Figure 4). It should be noted that several potential pathogens (e.g., *Acinetobacter venetianus*, *Brevundimonas naejangsanensis*, and *Pseudomonas marginalis*) were involved in vARG transfers, which may impose severe potential threats to the health of coastal citizens who frequently contact with estuarine water, especially with the fact that multidrug resistance genes were the most frequently transmitted vARGs.

The fate of ARGs in natural environments can be affected by several factors, including physicochemical properties (e.g., pH), microbial communities, and antibiotic concentrations [10, 55]. Here, we found that the richness, abundance, transmission, and expression of vARGs were greater in subtropical estuaries (Table S12) and showed significant positive correlations with temperature and human activities (Figures S9 and S10). Although the mechanisms that underlie these phenomena are not clear, we proposed some possible explanations. On the one hand, temperature is recognized as an important driver that influences microbial physiology, functions, and community compositions [56, 57]. Higher temperatures could increase prokaryotic metabolism and reproduction in estuaries, and in turn, boost viral replication and infection [58]. This increased viral activity may subsequently facilitate the proliferation, transmission, and expression of vARGs. On the other hand, population density and livestock production are generally higher in tropical and subtropical zones (with relatively higher temperatures) compared to other zones globally [59, 60]. Increased population density and livestock production could escalate antibiotic use [61], resulting in more antibiotic residues entering into the tropical/subtropical estuaries, which may increase the selective advantage of ARG‐carrying microorganisms and consequently facilitate the spread of vARGs [62]. Therefore, combining direct and indirect effects of increased temperature could amplify the ecological risks of vARGs, implying that the contribution of viruses to antibiotic resistome may be potentially elevated in the face of global warming (Figure 7). Although we have provided some potential explanations for the significant effects of temperature on the virus‐mediated ARG transfers, further evidence and controlled experiments are imperative to elucidate the underlying mechanisms. Moreover, the ability to investigate and quantify the drivers affecting vARG transmission and expression in estuarine sediments is currently constrained by insufficient data. Given the inherent complexity and dynamism of estuaries [63], a comprehensive collection of biotic and abiotic (e.g., physicochemical characteristics) parameters and long‐term monitoring of vARGs in estuaries is essential for thoroughly understanding the underlying drivers of vARGs.

**FIGURE 7:**
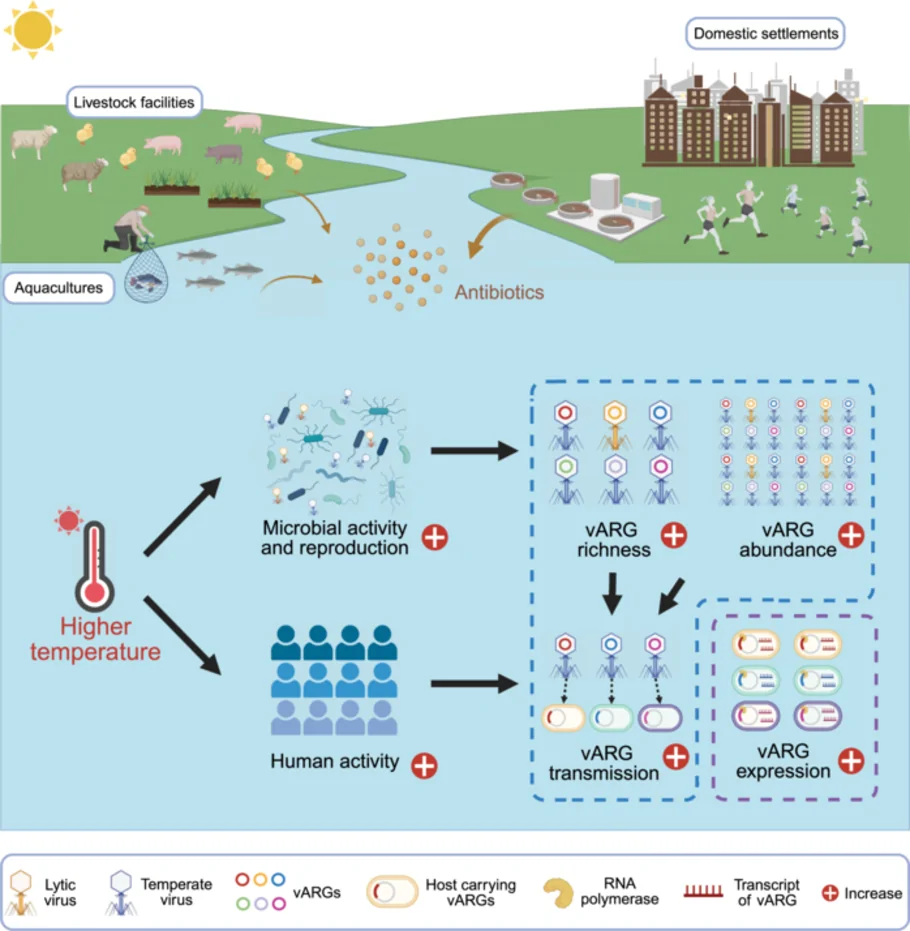
Conceptual model depicting the potential changes of vARG characteristics under global warming. Estuaries act as sinks of antibiotics that are mainly from coastal domestic settlements, livestock facilities, and aquaculture ponds. These antibiotics can exert strong selective pressure on estuarine microbes, resulting in diverse and abundant virus‐carried antibiotic resistance genes (vARGs) in estuaries. Higher temperatures would significantly increase the richness, abundance, transmission, and expression of vARGs via affecting human and microbial activities, suggesting that the contribution of viruses to estuarine antibiotic resistome may be potentially elevated in the face of global warming.

## 4 CONCLUSION

Our comprehensive analyses have revealed that despite ARG‐carrying viruses representing a small fraction of the estuarine vOTUs, they could (particularly temperate viruses) carry and express diverse ARGs, and facilitate the horizontal transfer of ARGs among abundant microbial taxa, including potential pathogenic species. Furthermore, increasing ambient temperature and coastal human activities may promote the richness, abundance, transmission, and expression of vARGs. Overall, our study presented the landscape of vARGs in global estuaries and highlighted their potential risks, suggesting the necessity of incorporating vARGs into future surveillance and management of estuaries, especially in the context of climate warming and social development.

## 5 MATERIALS AND METHODS

### Estuary sampling, sequencing, and physicochemical measurement

A total of 46 water samples and 14 sediment samples were taken from the Pearl River Estuary (PRE) during 2019–2020 according to previous methods [64, 65]. Specifically, for water samples, ~5 L surface water (~2–3 m depth) was sequentially filtered through a 3 μm (GSWP, Millipore) and 0.22 μm membrane filter (Pall Life Sciences) via a peristaltic pump to collect water microbes. For sediment samples, we collected surface sediments (0–20 cm) from 14 sites using a grab sampler. DNA and RNA extractions were performed simultaneously using standard methods as previously described [46]. After purifying the extracted DNA using the QIAquick Gel Extraction Kit (Qiagen), a DNA library was constructed using the NEBNext Ultra II DNA Prep Kit (New England Biolabs). Before constructing RNA library, the quality of all extracted RNA was detected by agarose gel electrophoresis, and those with DNA contamination were treated with DNase digestion. Moreover, ribosomal RNA was depleted in all extracted RNA with a Ribo‐Zero Magnetic Kit (Illumina) before library preparation. To accurately quantify gene expression levels for genes, all purified RNA was used for strand‐specific library preparation using the ALFA‐SEQ Directional RNA Lib Prep Kit (FINDROP). All DNA and RNA libraries were sent to the Magigene Company for metagenomic and metatranscriptomic high‐throughput sequencing on the Illumina NovaSeq. 6000 PE150 platform. Finally, we obtained a total of 60 metagenomes (46 water metagenomes and 14 sediment metagenomes) and 38 water metatranscriptomes.

Several physicochemical factors, including temperature, depth, dissolved oxygen, pH, salinity, turbidity, and chlorophyll a, were measured in situ by a conductivity‐temperature‐depth rosette system. As for the nutrient concentrations, we measured nitrate (NO_3_^−^), nitrite (NO_2_^−^), ammonia nitrogen (NH_4_^+^), total nitrogen (TN), phosphate (PO_4_^3−^), soluble reactive phosphate (SRP), sulfate (SO_4_^2−^), and total organic carbon (TOC) according to the procedures that have been described in detail by previous studies [66, 67].

### Estuarine data collection

In January 2023, we searched the NCBI SRA database using the keywords “estuary OR bay OR gulf OR coast OR lagoon” and obtained more than 2000 entries. After refining, 474 metagenomes and 150 metatranscriptomes (at least four million paired‐end reads per dataset) that were collected from estuarine environments were retrieved and downloaded. Combined with the sequencing data from the PRE, our final estuarine dataset contained a total of 534 metagenomes and 188 metatranscriptomes from 74 estuaries in nine countries, encompassing 551 water, 166 sediment, and 5 mat samples (Table S1).

Environmental variables, including locations and physicochemical factors (e.g., water depth, water temperature, salinity, turbidity, dissolved oxygen, pH, chlorophyll a, and nutrient concentration), of the published sequencing data were collected from their associated metadata (Table S1). River runoff of each estuary was estimated according to the annual hydrological survey (http://www.mwr.gov.cn/sj/; https://en.wikipedia.org/; Table S13). Additionally, climatic conditions (i.e., annual mean temperature and annual precipitation) and socio‐economic parameters (i.e., population density, urban ratio, gross domestic product per capita, meat and aquatic production, sewage emission, and number of patients diagnosed and treated) for each estuary were compiled based on governmental statistical yearbooks, bulletins and reports from nearby administrative regions [9] (Table S13). The selection of nearby regions was guided by the principle that if only specific districts/counties are situated within an estuarine basin, the data from these regions were collected at the district/county level; while if the entire or major part of a city is situated within the estuarine basin, the data from this region were collected at the city level. It is important to note that the socio‐economic data for estuaries in the USA were only available at the state level. Finally, all socio‐economic parameters (*S*) were normalized by population size according to Equation (1) to mitigate the influence of varying administrative region levels.

(1) $$S=\frac{\sum_{i=1}^{n}S_{i}}{\sum_{i=1}^{n}P_{i}},$$

where *n* is the number of districts/counties, cities, or states covered by the estuarine basin; *S*_*i*_ and *P*_*i*_ are the socio‐economic parameter and population size for each district/county, city, or state, respectively.

### Metagenome assembly

Raw metagenomic reads were first quality controlled using fastp v0.23.2 software [68] with parameters “‐n 0 ‐l 30 ‐5 ‐r ‐W 5 ‐cut_mean_quality 25 ‐‐detect_adapter_for_pe”. High‐quality reads of each metagenome were individually assembled using MEGAHIT v1.2.9 [69] with parameters “‐k‐min 27 ‐k‐max 127 ‐k‐step 20 ‐min‐contig‐len 500”. All assembled contigs with length > 3 kbp were combined and dereplicated by MMseqs2 v13.45111 [70] with parameters “‐‐min‐seq‐id 0.95 ‐c 0.95 ‐‐cov‐mode 1 ‐‐cluster‐mode 2.”

### Identification and classification of vOTUs

Four tools, including VIBRANT v1.2.1 [71], VirSorter2 v2.2.4 [72], DeepVirFinder v1.0 [73], and geNomad v1.8.1 [74] were utilized to identify putative viral contigs from the non‐redundant contigs with following criteria [36, 75]: (1) VIBRANT with default settings; (2) VirSorter2 with “‐‐high‐confidence‐only” parameter; (3) DeepVirFinder with score ≥0.9 and *p* < 0.05; (4) geNomad in conservative mode. Of all the putative viral contigs, those identified by two or more tools were considered as viral contigs, while the remaining contigs were further assessed to determine whether they were false positive viruses according to the number of bacterial and viral genes they carried [76]. Specifically, open reading frames (ORFs) of all remaining contigs were first predicted by Prodigal v2.6.3 [77] with “‐p meta ‐m ‐c.” Then, the ORFs were searched against bacterial universal single‐copy orthologues (BUSCO, odb10) [78] using Blastp v2.5.0+ with e‐value < 0.05 and score ≥ score‐cut‐offs identified by BUSCO [38, 76]. Viral genes were determined using Hmmsearch v3.3.2 against the curated viral protein family modules (VPFs) [79] with an e‐value < 0.05. The remaining contigs that had a BUSCO ratio <0.067 or had a BUSCO ratio ≥0.067 but with at least 10 VPF hits were considered as viral contigs [76]. To further remove the potential host‐derived contamination, all identified viral contigs were trimmed by CheckV v1.0.1 [80]. Then, only viral sequences with length ≥10 kbp were retained as previously suggested [81] and clustered into vOTUs using CD‐HIT‐EST v4.8.1 [82] with parameters “‐c 0.95 ‐aS 0.85.” Finally, we obtained 136,443 vOTUs forming the GEV database (Table S2). The longest contig of each vOTU was selected as the representative contig and used for subsequent vOTU‐related analyses.

Viral taxonomies were determined based on three methods: (1) geNomad [74] with conservative mode; (2) gene‐sharing network analysis using vConTACT2 v0.11.3 [83] against the Viral RefSeq r217 database [40]; (3) gene‐taxonomy‐based method [36], where a vOTU was assigned to a viral family if >50% of its Prodigal‐predicted ORFs exhibited best hits (Blastp v2.5.0+ [84], e‐value ≤ 1 × 10⁻⁵, bit score ≥50) to that family in the Viral RefSeq r217 database. Results from all methods were combined, and vOTUs with inconsistent assignments in different methods were manually corrected to “Unknown” to enhance accuracy.

To assess the genomic novelty of the GEV, vConTACT2 v0.11.3 [83] was used to determine the shared and specific vOTUs between the GEV and other databases, including GOV 2.0 dataset (GOV2.0, *n* = 195,728) [36], wetland sediment (*n* = 3344) [37], land use virome dataset (LVD, *n* = 61,106) [39] and Global Soil Virome dataset (GSV, *n* = 80,746) [38].

For lifestyle prediction, temperate viruses were conservatively confirmed by at least one of the three methods: (1) prophage signals identified by both VIBRANT v1.2.1 [71] and CheckV v1.0.1 [80]; (2) manual detection of lysogeny‐specific genes (i.e., integrase, recombinase, transposase, excisionase, CI/Cro repressor, and *parAB*) [85, 86]; (3) deep learning model using PhaTYP [87] (confident prediction score > 0.5). To detect the lysogeny‐specific genes, ORFs of each vOTU were annotated by searching against KEGG database using KofamScan v1.3.0 [88], eggNOG v5.0.0 using emapper v5.0.1 [89], COG (update in 2020) [90] and Viral RefSeq r217 database using DIAMOND v2.1.6.160 [91], Pfam v35.0 [92], VOG (release 202, http://vogdb.org/), the Prokaryotic Virus Orthologous Groups (pVOGs, release 5) databases [93], and the HMMs profiles from CheckV using Hmmsearch v3.3.2 [94]. The alignment criteria for all searches were set as “e‐value ≤ 10^−5^ and bit score ≥ 50.” vOTUs that did not contain any prophage signals or lysogeny features were classified as “virulent” or “unclassified” according to PhaTYP.

### Prokaryotic genome binning

Metagenome‐assembled genomes (MAGs) were recovered from the non‐redundant contigs according to the following pipeline. In brief, contigs were binned into MAGs based on the coverage variation of contigs across samples and tetranucleotide frequencies using MetaBAT2 v2.14 [95] with default parameters. The potential contaminations were identified and removed by RefineM v0.1.2 [96], and the viral contigs that “mis‐binned” with MAGs were manually removed. The qualities of refined MAGs were evaluated by CheckM2 v1.0.1 [97]. MAGs with Medium‐to‐High quality (completeness > 60%, contamination < 5%) were then dereplicated by dRep v2.6.2 [98] with 99% average nucleotide identity. The taxonomy of each MAG was identified by GTDB‐Tk v2.3.0 [99].

### Reconstruction of viral and prokaryotic communities

To calculate the relative abundance (calculated as Reads Per Kilobase per Million mapped reads, RPKM) of each vOTU, high‐quality metagenomic reads from each sample were mapped to the GEV using Bowtie2 v2.4.5 [100] with default parameters. All mapping files were then passed to CoverM v0.5.0 [39] to generate abundance profiles across samples after removing low‐quality mapping reads with parameters “‐‐min‐read‐percent‐identity 95 ‐‐min‐read‐aligned‐percent 95 ‐‐min‐covered‐fraction 0.6”. As for prokaryotic communities, the taxonomies of non‐viral contigs were first assigned using MMseqs2 v13.45111 [70] against Genome Taxonomy Database (GTDB) r214 [101]. Only contigs that were classified as “Bacteria” and “Archaea” were regarded as prokaryotic sequences. Subsequently, the relative abundance (RPKM) of each prokaryotic contig was computed using the same method as aforementioned.

### ARG annotation and abundance

To perform ARG annotation, we first compiled a comprehensive ARG database by combining CARD v3.2.2 (only proteins in homolog model) [102], SARG v2.0 [103], Resfam v1.2 (core database) [104], ARGMiner [105] and ARG RefSeq proteins (https://www.ncbi.nlm.nih.gov/pathogens/refgene/), resulting in 26,180 non‐redundant ARG protein sequences after clustering at 100% nucleotide identity using CD‐HIT v4.8.1 [82]. Then, ARGs carried by viral and prokaryotic sequences were identified by searching against the integrated ARG database using Blastp v2.5.0+ with conservative criteria (≥70% identity and ≥70% coverage) [106]. Given that the high sequence similarity will largely only identify known ARGs, we further inferred ARGs from Resfam core database using Hmmsearch v3.3.2 with “‐‐cut‐ga” as previously suggested [106], and refined their classification levels by querying against the CARD database with the top blastp hit. It should be noted that the virus‐encoded ARGs (vARGs) were only identified within viral conserved regions [107], where both the start and end genes were annotated as viral hallmarks or viral proteins, which were identified by searching against the VPFs and viral‐specific HMMs profiles from CheckV's database using Hmmsearch v3.3.2 with criteria “e‐value ≤ 10^−5^ and bit score ≥ 50”. Moreover, to further improve the reliability of functional annotation, predicted ARGs in viral conserved regions were further filtered based on their 3D protein structure using Phyre2 [108] with a rigorous parameter “100% confident and ≥70% alignment [107]” (e.g., for predicted efflux pumps, only those identified as “drug transporter/multidrug efflux pump” by Phyre2 were retained). Same procedures of vARG annotation were also performed for the GOV 2.0 datasets. Given that most vARGs are host‐derived, we represented the abundances of vARGs and pARGs using the abundances of corresponding contigs that carried these ARGs to ensure that the abundance values were unambiguously attributed to viruses or prokaryotes [86].

### Virus‐host linkage prediction

To predict the viral hosts, we first constructed a prokaryotic host reference genome database by including 1340 medium‐to‐high quality MAGs recovered from our dataset and all representative genomes of aquatic microbes (defined as genera that inhabit the estuarine/freshwater/marine/sedimentary/aquatic environments as previously reported [109]) retrieved from the GTDB r214 database. We then predicted virus‐host linkages using three methods [76]. (1) *Prophage blasts*: Blastn v2.5.0+ was used to align vOTUs to the host reference database with parameters “identity ≥ 90%, query coverage ≥75%, e‐value ≤ 10^−3^, bit score ≥ 50” as previously suggested [110]. These matches were then further filtered by host contig coverage (only matches with a matched region <70% length of the host contig were retained) to avoid mis‐binned viral fragments in the host metagenome assembled genome. (2) *tRNA match*: tRNAs in vOTUs were identified by tRNAScan‐SE v1.23 [111] in bacterial and archaeal modes, and all extracted tRNAs that matched promiscuous tRNAs from the Earth virome dataset [112] were removed from further analyses. The filtered tRNAs were then queried against the host reference database using Blastn v2.5.0+ with parameters “identity = 100%, coverage = 100%”. (3) *CRISPR spacers match*: the CRISPR spacers were first extracted from the host reference database by MinCED v0.4.2 [113] with default parameters, and then queried against all vOTUs using Blastn v2.5.0+ with parameters “word size = 16, coverage = 100%, mismatch ≤ 1, e‐value ≤ 10^−6^ [114]. For all virus‐host CRISPR spacer match pairs, only those with one exact (mismatch = 0) spacer match or multiple (≥ 2) spacer matches (each with mismatch = 1) were retained. Finally, the hosts predicted by any of the methods were combined as the final potential hosts of viruses. All predicted virus–host linkages are available in Table S6.

### ARG transfers between viruses and their hosts

Given the independent evolution of viral genes and their host homologues (e.g., viral *amoC* genes formed a distinct phylogenetic cluster comparing with bacterial *amoC* genes [115]), metagenomics cannot reliably identify ancient horizontal gene transfer events between viruses and their hosts. Therefore, we employed the minimum evolutionary distance method to conservatively investigate recent transfer events [32]. Briefly, the predicted prophage sequences and mis‐binned viral fragments were first temporarily removed from their host MAGs to minimize the potential bias caused by the virus‐derived ARGs. ARGs carried by the bona fide host genomes were identified using blastp and Hmmsearch as described in the section “ARG annotation and abundance.” Subsequently, all ARGs carried by vOTUs and host MAGs were clustered into homologous protein clusters (HpCs) using CD‐HIT v4.8.1 [82] with parameters “‐c 0.9 ‐n 5 ‐g 1 ‐aS 0.9.” For HpCs containing both virus and host‐derived ARGs, multiple sequence alignments were performed using MUSCLE v5.1 [116], and the resulting alignments were trimmed by trimAl v1.5 (“‐strictplus” option) [117]. Subsequently, pairwise evolutionary distances were computed using PHYLIP‐protdist v3.69 (https://phylipweb.github.io/phylip/doc/protdist.html) with default settings [32]. Finally, a potential virus‐host ARG transfer event was identified according to the minimal evolutionary distance and virus‐host linkages within each HpC. All virus‐host ARG transfers were visualized using Cytoscape software (version 3.2.1) [118].

### Expression of vARGs and pARGs

The expression profiles of vARGs and pARGs were computed as Transcripts Per kilobase Million by mapping high‐quality metatranscriptomic reads to all ARG sequences (coding strand) using Bowtie2 v2.4.5 [100] and applying a strict filtering criterion (100% identity and 100% coverage of short reads) via CoverM v0.5.0 [119]. It should be noted that ARG sequences that were identical (100% identity with 100% coverage) between vARGs and pARGs were excluded from the final expression profiles to minimize the potential bias caused by ambiguous mapping. In total, 89 vARGs and 107 pARGs were removed. The relative contribution of vARG expression within each sample was determined by dividing the expression abundance of vARGs by the total expression abundance of both vARGs and pARGs [120].

### Statistical analyses

All statistical analyses were performed using R software (version 4.3.1). Non‐metric multidimensional scaling (NMDS) was conducted to visualize the separation of viral communities (relative abundances of vOTUs) and vARG compositions (relative abundances of ARG subtypes) of different samples based on the Bray‐Curtis distance. The differences among countries were verified by ANOSIM analysis with 999 permutations. It should be noted that the samples from the Netherlands, Sweden, and Thailand were excluded from the NMDS analysis due to a lack of raw reads or no vOTU detection (with at least 60% coverage mapping). The richness of vARGs was calculated at the ARG subtype level using “diversity” function in *vegan* package. The significance of differences in vARG richness and abundance between habitats was assessed using the Mann‐Whitney *U* test, whereas differences among countries were evaluated with a Kruskal‐Wallis test, followed by Dunn's post hoc test with Benjamini‐Hochberg adjustment. Additionally, a univariate logistic regression model was employed to examine the relationship between vARG density (number of vARGs per kbp of each vOTU) and host range (two categories: hosts belonged to one taxon or ≥ 2 taxa at the genus, family, or order levels).

To explore the features of ARG transfers in each sample, we generated sub‐networks from the whole ARG transfer network by preserving vOTUs and host MAGs presented in each sample using “subgraph” function of *igraph* package, and calculated the network‐level topological features, including number of edges (number of vARG transfer types) and average path length (transfer range of vARG). Additionally, the potential risk of virus‐mediated ARG transfers (RI), which represents the diversity of vARG transfers, was calculated using Equation (2).

(2) $$\mathrm{RI}={\left(\sum_{i=1}^{n}A_{i}^{2}\right)}^{-1},$$

where *n* is the total number of edges in each sample, and *A*_*i*_ is the proportional abundance of the *i*th edge, which was calculated as the product of the abundances of vOTU and its host linked by the *i*th edge. The square of *A*_*i*_ indicates greater sensitivity of the true diversity to abundant ARG transfers. However, sediment samples were excluded from this analysis due to their limited number of vARG transfers.

For exploring how variations in the whole viral communities and vARG characteristics (i.e., vARG composition, richness, abundance, transmission, and expression) were linked to environmental conditions and the prokaryotic assemblages, Spearman's rank correlation coefficient (*ρ*; two‐tailed) were calculated to reveal the pairwise relationships among all biotic and abiotic factors. Bray‐Curtis distances were calculated for viral and prokaryotic community structures and vARG compositions, whereas Euclidean distances were calculated for environmental variables. Mantel tests with 999 permutations were performed to identify the correlations between these dissimilarity matrices. It should be noted that some physicochemical factors (i.e., depth, TN, and SO_4_^2−^) with missing values in > 80% of the samples were excluded from the correlation analyses (Table S1). For the remaining physicochemical factors, pairwise deletion was applied to handle incomplete data. In all correlation analyses, *p* values were adjusted for multiple testing using the Benjamini‐Hochberg false discovery rate controlling procedure (*q* < 0.05). Notably, considering the potential impact of low or unequal sequencing depths on vARG richness, we repeated the correlation analysis after filtering samples with less than 20 million reads (301 samples were kept) or normalizing the vARG richness by reads number per sample. Similar results were obtained, suggesting that sequencing depth had a limited influence on the results (Figure S13).

Furthermore, based on the correlation results, six environmental variables (latitude, temperature, precipitation, population density, urban ratio, and GDP per capita), along with all biotic variables, were selected to explain the variations of vARG characteristics using random forest analysis. Only predictors among the top ten of importance (%IncMSE) were included in structural equation modeling (SEM, *piecewiseSEM* v2.3.0 packages). Specifically, priori models were first constructed by considering all potential mechanisms, where environmental factors influence prokaryotic and viral communities, as well as pARGs, and in turn the vARGs' characteristics. Then, we refined the SEM by removing nonsignificant paths and adding missing paths. The overall fit of a piecewise SEM is evaluated using Shipley's d‐separation test (good model fitness: *p* > 0.05) [121].

## 6 AI STATEMENT

The initial draft of this manuscript was polished for grammar and sentence structure using DeepSeek (R1, DeepSeek Inc.) and Grammarly (https://app.grammarly.com/). All AI‐generated contents were meticulously reviewed and edited by the authors to ensure factual accuracy and prevent any potential AI‐induced bias. We hereby confirm that no AI tools were employed in the processes of data generation, analyses, or visual content creation.

## AUTHOR CONTRIBUTIONS

**Xiao‑Qing Luo**: Conceptualization; methodology; data curation; writing—original draft; writing—review & editing; formal analysis; visualization; validation; investigation. **Xiyang Dong**: Conceptualization; writing—review & editing; methodology. **Jia‑Ling Li**: Data curation. **Ji‐Cheng Yao**: Data curation. **Ziqi Peng**: Data curation. **Qi Li**: Data curation. **Zi‐Wen Yang**: Data curation. **Yang Yuan**: Data curation. **Wen‑Jun Li**: Funding acquisition; conceptualization; writing—review & editing; supervision; project administration; resources. **Pandeng Wang**: Conceptualization; methodology; data curation; formal analysis; writing—review & editing; funding acquisition; supervision; resources. All authors have read the final manuscript and approved it for publication.

## CONFLICTS OF INTEREST STATEMENT

The authors declare no conflicts of interest.

## ETHICS STATEMENTS

No animals or humans were involved in this study.

## Supporting information


**Figure S1.** Bioinformatic workflow.
**Figure S2.** Niche‐specific distributions of vOTUs in the GEV.
**Figure S3.** Drivers of prokaryotic and viral community compositions in estuarine water (A) and sediment (B).
**Figure S4.** Changes of the relative proportion of different viral lifestyles in ARG‐carrying viruses across different samples.
**Figure S5.** Comparisons of the richness and total abundance of vARGs between water and sediment habitats.
**Figure S6.** Geographical distribution of vARG richness and abundance.
**Figure S7.** Comparisons of the richness and total abundance of vARGs among different countries.
**Figure S8.** Changes of vARG compositions across different countries.
**Figure S9.** Drivers of vARG richness, abundance, and transmission in estuarine water (A) and sediment (B).
**Figure S10.** Drivers of vARG expression in estuarine water.
**Figure S11.** Drivers of vARG richness and abundance in estuarine sediment.
**Figure S12.** Changes of the relative proportion of active vARGs from different viral lifestyles across samples.
**Figure S13.** Drivers of vARG richness in estuarine water and sediment with rarefied datasets.

**Table S1.** Detailed information of the estuarine samples.
**Table S2.** Detailed information of all vOTUs in the global estuarine virome (GEV) dataset.
**Table S3.** Detailed information of ARG‐carrying vOTUs in the GEV.
**Table S4.** Relative proportion of ARG‐carrying viruses in Global Ocean Viromes (GOV) dataset.
**Table S5.** Functional annotation of the virus‐carried ARGs (vARGs) in the GEV.
**Table S6.** Predicted virus‐host linkages in the GEV.
**Table S7.** Logistic regression analyzing the relationship between vARG densities and their host ranges.
**Table S8.** Potential ARG transfers between viruses and their predicted hosts.
**Table S9.** Expression (TPM) of vARGs in all estuarine samples.
**Table S10.** Total expression abundances (TPM) of vARGs and prokaryote‐carried ARGs (pARGs) in the estuaries.
**Table S11.** Expression (TPM) of viral and prokaryotic ARG subtypes in estuaries.
**Table S12.** Average vARG richness and abundance in various estuaries.
**Table S13.** Social‐economic data collected according to the administrative regions that nearby the estuaries.

## Data Availability

In‐house sequencing data generated from the Pearl River Estuary are deposited in the NCBI Sequence Read Archive (SRA) database and the China National Center for Bioinformation (CNCB; https://ngdc.cncb.ac.cn/). The accession numbers and URLs for downloaded sequencing data from the NCBI SRA database and in‐house sequencing data are listed in Table S1. The bioinformatic workflow, R scripts, and relevant data used to generate figures for this study are provided within this paper and publicly available from https://doi.org/10.6084/m9.figshare.30496010. Supplementary materials (results, figures, tables, graphical abstract, slides, videos, Chinese translated version, and update materials) may be found in the online DOI or iMetaOmics http://www.imeta.science/imetaomics/.
