# Supplementary material for: Diversity, transfer potential, and transcriptional activity of virus‐carried antibiotic resistance genes in global estuaries
Source: IMetaOmics. 2026 Jan 7;3(1):e70074. doi: 10.1002/imo2.70074 (PMC13674435; doi:10.1002/imo2.70074)
Supplement: Supplementary file 1 [file IMO2-3-e70074-s001.docx]

Supporting information to

**Diversity, transfer potential, and transcriptional activity of virus-carried antibiotic resistance genes in global estuaries**

Running title: Virus-carried antibiotic resistance genes in global estuaries

Xiao‑Qing Luo^1,2,#^, Xiyang Dong^3,#^, Jia‑Ling Li^4^, Ji-Cheng Yao^5^, Ziqi Peng^2^, Qi Li^2^, Zi-Wen Yang^2^, Yang Yuan^5^, Wen‑Jun Li^2,*^ and Pandeng Wang^1,*^

^1^ School of Pharmacy (Shenzhen University Medical School), College of Life Sciences and Oceanography, and Institute for Advanced Study, Shenzhen University, Shenzhen 518060, China

^2^ State Key Laboratory of Biocontrol, Guangdong Provincial Key Laboratory of Plant Stress Biology, School of Life Sciences, Sun Yat-Sen University, Guangzhou 510275, China

^3^ Key Laboratory of Marine Genetic Resources, Third Institute of Oceanography, Ministry of Natural Resources, Xiamen 361005, China

^4^ Southern Marine Science and Engineering Guangdong Laboratory (Guangzhou), Guangzhou 511458, China

^5^ Institute of Marine Biotechnology and Bio-Resource Utilization, College of Oceanography, Hohai University, Nanjing 210098, China

\# These authors contributed equally: Xiao‑Qing Luo, Xiyang Dong

* Correspondence:

wangpd@mail2.sysu.edu.cn (Pandeng Wang);

liwenjun3@mail.sysu.edu.cn (Wen‑Jun Li)

# Supplementary results

## Spatial distribution of estuarine virus-carried ARGs (vARGs)

Estuarine vARGs exhibited remarkable spatial heterogeneity. Specifically, the richness of vARGs in water was significantly greater than that in sediment (**Figure S5**), suggesting that microbes inhabiting water bodies may be experiencing greater selection pressure from antibiotics because most antibiotics are water-soluble [1, 2]. Additionally, the vARG richness varied significantly among estuaries of different countries (**Figure S6 and Figure S7**). Within water fraction, we observed the greatest richness of vARGs in Pearl River Estuary and Chilika Lagoon with an average of over 10 ARG subtypes per sample, closely followed by the Yangtze River Estuary with an average of 8 ARG subtypes per sample (**Table S12**). While in sediment, Victoria Harbor exhibited the greatest vARG richness (average 7 ARG subtypes per sample), followed by the Gulf of Khambhat (average 3 ARG subtypes per sample, **Table S12**). Inconsistent with the findings of vARG richness, the abundances of vARGs showed no significant difference between water and sediment (**Figure S5**). Furthermore, the abundances of water vARGs were highest in Chinese estuaries, particularly in the Pearl River Estuary and the Moyangjiang River Estuary (**Figure S7 and Table S12**). Regarding estuarine sediment, vARGs in Canada exhibited greater abundances than those in China (**Figure S7**).

The profiles of vARGs also exhibited significant distinction across different habitats and countries (**Figure S8**), though they were mostly predominated by nitroimidazole and multidrug-resistant genes, which coincided with the habitat-dependent viral community compositions (**Figure 1E**). Specifically, vARGs conferring resistance to nitroimidazole and Tetracycline were commonly enriched in estuarine water column, and the relative abundances of quinolone, phenol, and trimethoprim resistance genes were greater in Chinese estuaries. Contrasting, estuarine sediments were characterized by abundant vARGs associated with aminocoumarin resistance, and a relatively high proportion of vARGs conferring resistance to MLS and peptide antibiotics in Canada.

# Supplementary figures

**Figure S1 Bioinformatic workflow.** Arrows illustrate the order of computational procedures, encompassing **(A)** estuarine data collection and metagenomic assembly, **(B)** classification of prokaryotic and viral sequences from metagenome-assembled contigs, and construction of the global estuarine virome (GEV) dataset, **(C)** gene prediction, functional annotation and abundance calculation, **(D)** determination of host assignment, **(E)** spatial distribution of virus-carried ARGs (vARGs), **(F)** construction of virus-mediated ARG transfer network, and **(G)** revealing the driving factors of vARG richness, abundance, dissemination and expression. The red text under (or beside) arrows highlights the software used in a specific program.

**Figure S2 Niche-specific distributions of vOTUs in the GEV.** **(A)** Venn diagram displaying the number of unique and shared vOTUs in water and sediment habitats after rarefying the water samples. **(B)** Venn diagrams show the number of unique and shared vOTUs across different countries in water and sediment habitats. **(C)** Bars denote the relative abundance of taxonomy-annotated vOTUs (family-level) in estuarine water and sediment.

**Figure S3 Drivers of prokaryotic and viral community compositions in estuarine water (A) and sediment (B).** The color gradient denotes Spearman’s rank correlation coefficient, and the asterisk indicates Benjamini-Hochberg adjusted statistical significance, * *q* < 0.05, ** *q* < 0.01, and *** *q* < 0.001. Viral and prokaryotic community compositions were related to each variable by Mantel tests. Edge width corresponds to Mantel’s *r* statistic for the corresponding distance correlation, and edge color denotes the statistical significance. DO, dissolved oxygen; Chla, chlorophyll a; ORP, oxidation-reduction potential; GDP, gross domestic product per capita; TOC, total organic carbon; S, sulfur.

**Figure S4 Changes of the relative proportion of different viral lifestyles in ARG-carrying viruses across different samples.** The relative proportions of different viral lifestyles were calculated by dividing the number of ARG-carrying viruses that adopt specific lifestyle by the total number of ARG-carrying viruses in each sample.

**Figure S5 Comparisons of the richness and total abundance of vARGs between water and sediment habitats.** The comparisons of vARG richness and abundance between habitats were conducted for both the global estuarine dataset **(A)** and a rarefied estuarine dataset **(B)**, in which water samples were rarefied to the same sample number as sediment samples. The significance of the differences was determined by the Mann-Whitney *U* test (* *p* < 0.05 and ** *p* < 0.01).

**Figure S6 Geographical distribution of vARG richness and abundance.** The maps show the geographical distribution of vARG richness and abundance in water and sediment samples. Dot color and size denote the average richness and abundance of vARGs in each estuary, respectively.

**Figure S7 Comparisons of the richness and total abundance of vARGs among different countries.** Different letters above the boxes indicate statistically significant differences (Dunn’s test, Benjamini-Hochberg adjusted, *q* < 0.05) between pairwise countries. Groups sharing the same letter are not significantly different.

**Figure S8 Changes of vARG compositions across different countries.** Non-metric multidimensional scaling (NMDS) of vARG compositions in water **(A)** and sediment **(B)** based on Bray-Curtis dissimilarity. The significant differences of vARG compositions among countries were verified by analysis of similarities (ANOSIM; 999 permutations). **(C)** Bar charts show the compositions of vARGs in different samples. CH, China; I, India; CA, Canada.

**Figure S9 Drivers of vARG richness, abundance, and transmission in estuarine water (A) and sediment (B).** The color gradient denotes Spearman’s rank correlation coefficient, and the asterisks represent Benjamini-Hochberg adjusted statistical significance, * *q* < 0.05, ** *q* < 0.01, and *** *q* < 0.001. Edge width corresponds to Mantel’s *r* statistic for the corresponding distance correlations, and edge color denotes the statistical significance. DO, dissolved oxygen; Chla, chlorophyll a; ORP, oxidation-reduction potential; GDP, gross domestic product per capita; TOC, total organic carbon; S, sulfur; Prokaryotic, the whole prokaryotic community; Viral, the whole viral community; ARG frequency, the number of ARG-carrying vOTUs divided by the total number of vOTUs presented in each sample.

**Figure S10 Drivers of vARG expression in estuarine water.** The color gradient denotes Spearman’s rank correlation coefficient, and the asterisks represent Benjamini-Hochberg adjusted statistical significance, * *q* < 0.05, ** *q* < 0.01, and *** *q* < 0.001. vARG expression, total expression abundance (TPM value) of vARGs in each sample; pARG expression, total expression abundance of prokaryotic-carried ARGs (pARGs) in each sample; Expression ratio, vARG expression divided by pARG expression; Relative expression, the ratio of vARG expression to the sum expression of all vARGs and pARGs; GDP, gross domestic product per capita.

**Figure S11 Drivers of vARG richness and abundance in estuarine sediment.** **(A)** Random forest analysis determined the relative importance of variables (only showing the top nine) influencing vARG richness and abundance. R^2^ (pseudo) represents the total variance that could be explained by the selected environmental variables. pTax, the whole prokaryotic communities; vTax, the whole viral communities. **(B)** The structural equation model shows the direct and indirect effects of biotic and abiotic factors on the richness and abundance of vARGs. Black arrows denote positive associations. The standardized path coefficients (*r*, black number) are shown along the paths, and R^2^ (orange number) represents the explained variance for every dependent variable. Temp, air temperature; R, richness; A, abundance.

**Figure S12 Changes of the relative proportion of active vARGs** **from different viral lifestyles across samples.** The relative proportions of active vARGs from different viral lifestyles were determined by dividing the number of active vARGs from viruses with a specific lifestyle by the total number of active vARGs in each sample.

**Figure S13 Drivers of vARG richness in estuarine water and sediment with rarefied datasets.** Correlations between environmental variables and vARG richness in rarefied datasets, including **(A)** removed samples with sequencing depths below 20 million reads and **(B)** normalized the vARG richness by reads number per sample. The color gradient denotes Spearman’s rank correlation coefficient, and the asterisks represent Benjamini-Hochberg adjusted statistical significance, * *q* < 0.05, ** *q* < 0.01, and *** *q* < 0.001. Edge width corresponds to Mantel’s *r* statistic for the corresponding distance correlation, and edge color denotes the statistical significance. DO, dissolved oxygen; Chla, chlorophyll a; ORP, oxidation-reduction potential; GDP, gross domestic product per capita; TOC, total organic carbon; S, sulfur; Prokaryotic, the whole prokaryotic community; Viral, the whole viral community; ARG frequency, the number of ARG-carrying vOTUs divided by the total number of vOTUs presented in each sample.

# References

1. Jiang, Haoyu, Renjun Zhou, Mengdi Zhang, Zhineng Cheng, Jun Li, Gan Zhang, Baowei Chen, Shichun Zou, Ying Yang. 2018. “Exploring the differences of antibiotic resistance genes profiles between river surface water and sediments using metagenomic approach.” *Ecotoxicology and Environmental Safety* 161: 64-69. <https://doi.org/10.1016/j.ecoenv.2018.05.044>

2. Wu, Yang, Si Li, Ke Yu, Jingrun Hu, Qian Chen, Weiling Sun. 2023. “Wastewater treatment plant effluents exert different impacts on antibiotic resistome in water and sediment of the receiving river: Metagenomic analysis and risk assessment.” *Journal of Hazardous Materials* 460: 132528. <https://doi.org/10.1016/j.jhazmat.2023.132528>
